# Localized In Situ Nanoemulgel Drug Delivery System of Quercetin for Periodontitis: Development and Computational Simulations

**DOI:** 10.3390/molecules23061363

**Published:** 2018-06-04

**Authors:** Gururaj C. Aithal, Usha Yogendra Nayak, Chetan Mehta, Reema Narayan, Pratibha Gopalkrishna, Sudharsan Pandiyan, Sanjay Garg

**Affiliations:** 1 Department of Pharmaceutics, Manipal College of Pharmaceutical Sciences, Manipal Academy of Higher Education, Manipal 576104, India; gururajaithal262@gmail.com (G.C.A.); chetan_jd12@rediffmail.com (C.M.); nsreema@gmail.com (R.N.); 2 Department of Periodontology, Manipal College of Dental Sciences, Manipal Academy of Higher Education, Manipal 576104, India; pratibha.pk@manipal.edu; 3 Schrödinger, Bengaluru, Karnataka 560086, India; sudharsan.pandiyan@schrodinger.com; 4 School of Pharmacy and Medical Science, University of South Australia, Adelaide, SA 5000, Australia; Sanjay.Garg@unisa.edu.au

**Keywords:** nanoemulgel, periodontitis, spontaneous emulsification, antimicrobial, molecular simulation, computational formulation design

## Abstract

This study was aimed at formulating a bioabsorbable, controlled-release, nanoemulgel of Quercetin, a potent antimicrobial and anti-inflammatory agent for the treatment of periodontitis that could improve its solubility and bioavailability. Screening of components was carried out based on the solubility studies. Nanoemulsion containing cinnamon oil as the oil phase, tween 80 and Carbitol^®^ as the surfactant-cosurfactant mixture (S_mix_) and water as the aqueous phase containing 125 µg/200 µL of Quercetin was prepared by using spontaneous emulsification method. Nanoemulgel was prepared using 23% *w*/*v* poloxamer 407 as gel base. Comprehensive evaluation of the formulated nanoemulgel was carried out, and the optimized formulation was studied for drug release using Franz vertical diffusion cells. The formulated nanoemulgelexhibited a remarkable release of 92.4% of Quercetin at the end of 6 h, as compared to that of pure Quercetin-loaded gel (<3% release). The viscosity of the prepared nanoemulgel was found to be 30,647 ± 0.32 cPs at 37 °C. Also, molecular dynamics (MD) simulation was utilized to understand the gelation process and role of each component in the formulation. The present study revealed that the developed nanoemulgel of Quercetin could be a potential delivery system for clinical testing in periodontitis.

## 1. Introduction

Periodontitis is a chronic inflammatory disease affecting the supporting structures of the teeth. It is a disease of multifactorial etiology, with microbial, genetic, environmental and host factors involved. As a result, a state of chronic inflammation persists, with the release of oxygen-free radicals by the inflammatory cells, mainly the polymorphonuclear lymphocytes. Inadequate dental plaque removal results in the inflammation of gums/gingivae and their separation from the tooth surface by marginal tissue recession and deepening of the gingival sulcus resulting in periodontal pocket formation. This process is accompanied by destruction of tooth-supporting bone, thereby compromising the support of the tooth and its eventual exfoliation. This destructive process is facilitated by the enzymes and toxic by-products released by the periodontal pathogenic microflora, which can break down host cell membranes and extracellular matrices, to produce nutrients essential for their growth [1,2,3,4,5]. Periodontal disease is currently being investigated for association with a large number of systemic diseases like cardiovascular disease, rheumatoid arthritis, and diabetes [6,7,8].

Therapeutic management of the disease includes nonsurgical treatment of periodontal disease with scaling and root planing, systemic antibiotics, anti-inflammatory and bone regenerating agents, etc. Various antimicrobial agents have also been tested in local delivery systems, for their efficacy in the inhibition of colonization as well as in the prevention of plaque formation [9]. Non-steroidal anti-inflammatory drugs (NSAIDs) have also reportedly been used with limited success [10]. Nevertheless, orally administered drugs suffer from drawbacks due to their systemic effect and also result in lack of effective concentration of the drug at the site of action, resulting in poor patient acceptance. This necessitates the development of alternative localized delivery of drugs. Many in situ localized deliveries of drugs such as simvastatin, atorvastatin, ornidazole, levofloxacin, and flurbiprofen have been evaluated with positive results for periodontitis [11,12,13,14,15].

Recent studies have reported that phytochemicals have been found to be effective in the treatment of periodontitis [16]. Bioflavonoids, particularly Quercetin, have shown potential antimicrobial activity, lowering of inflammatory markers, cholesterol reduction and inhibiting bone loss. However, this data has largely been obtained from in vitro and animal studies, but data from human studies are limited. Quercetin has been found to decrease the extracellular matrix degradation, promote wound healing when tested in gingival fibroblasts, and showed excellent antibacterial properties. It also possesses anti-inflammatory effects which can be explored to restrict the inflammation in periodontitis [17,18]. Hence, in the present study, we attempted to formulate an in situ nanoemulgel of Quercetin based on the antimicrobial sensitivity to specific periodontal pathogens [19,20,21]. Despite the beneficial effects of Quercetin, its poor aqueous solubility and poor bioavailability result in limited absorption. Scientists have worked on improving the bioavailability of Quercetin using various approaches, such as nanosuspension [22], self-nano emulsifying systems [20], microemulsion [23], solid lipid nanoparticles [24], cyclodextrin complexes [25]. The combination of oils and emulsifiers enhances the absorption of Quercetin [26]. Hence, nanoemulsion could be a suitable drug delivery vehicle for the loading of Quercetin. Nanoemulsions are more beneficial than microemulsions, as they have high kinetic stability and a smaller droplet size [27]. Nanoemulsion increases the rate of absorption and eliminates variability in absorption, helps in solubilizing lipophilic drug and increases bioavailability. It allows rapid and efficient penetration of the drug moiety. These can be incorporated into the polymer solution to form in situ nanoemulgel, which provides sustained/controlled drug delivery and ease of administration, thus enhancing patient compliance.

Hence, in the present study, the advantages of nanoemulgel were exploited for the efficient delivery of Quercetin. Also, a computer-aided formulation design approach was used to understand the gelation process and role of each component in the formulation. Computer-aided formulation design approaches are the current trend in the context of dosage form development. The computational approach will not only reduce the experimental efforts, but also help in studying the mechanistic physiological behavior of the dosage form [28,29].

## 2. Results and Discussion

### 2.1. Screening of Nanoemulsion Components Based on the Solubility of Quercetin

The solubility of the drug in oil plays a major role in formulating nanoemulsion to incorporate the maximum dose and maintain the drug in solubilized state. Therefore, the components exhibiting the highest solubility were utilized in different combinations in conducting formulation trials to obtain a nanoemulsion. On the basis of the saturation solubility, the components exhibiting the highest solubility were categorized according to the order of their solubility. The order of Quercetin solubility in oils was cinnamon oil > triacetin > castor oil > sesame oil > labrafac > palm oil > isopropyl myristate > migliyol. The solubility order in surfactants was labrasol > tween 80 > tween 60 > cremophor EL > span 80 > tween 20 and cosurfactants was transcutol HP > carbitol > ethylene glycol > ethanol > capryol > lutrol 400 > lutrol 600 > glycerol (Refer Supplementary Materials Figures S1 and S2).

### 2.2. Construction of Pseudo-Ternary Phase Diagrams

**Figure 1:**
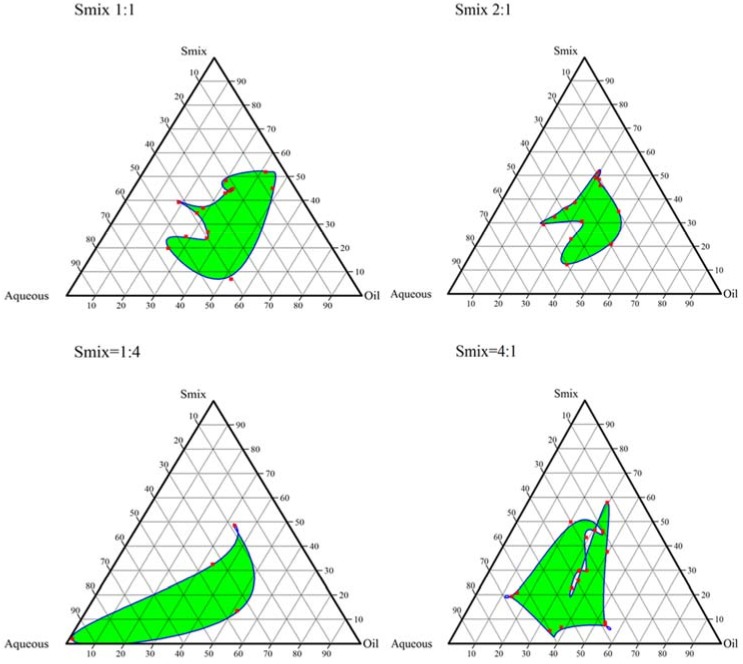
Ternary plots for nanoemulsions obtained using cinnamon oil, tween 80 and ethylene glycol.

**Figure 2:**
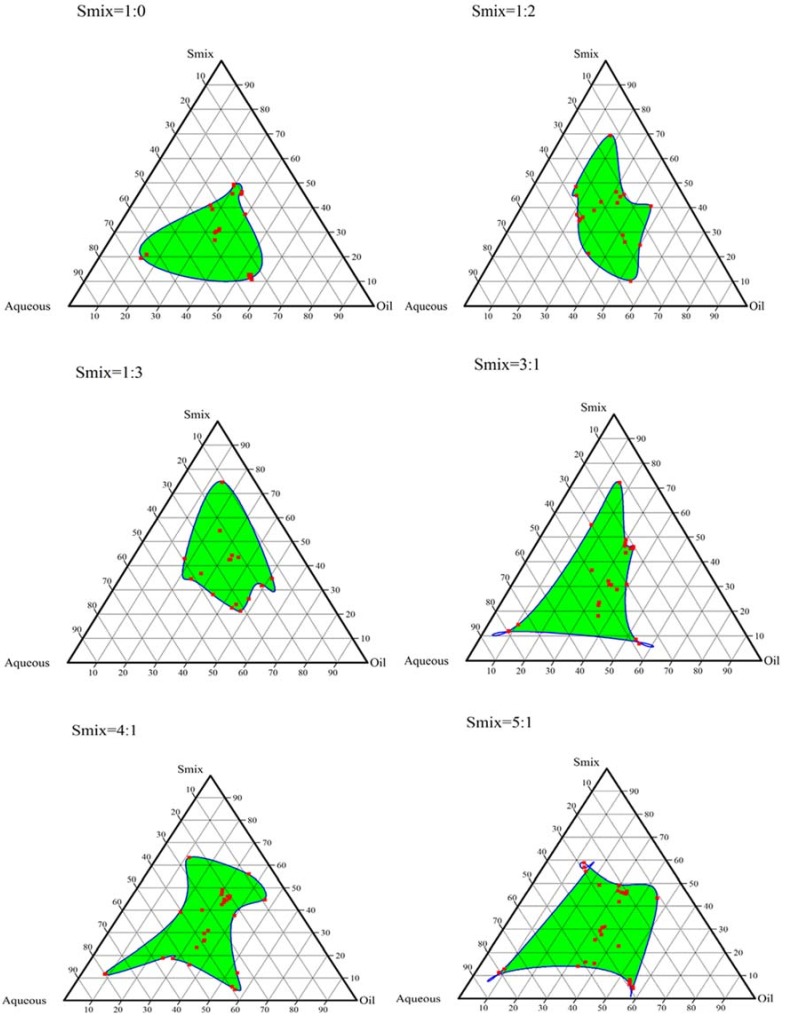
Ternary plots for nanoemulsions obtained using cinnamon oil, tween 80 and Carbitol^®^.

Based on the saturation solubility study, different formulation trials were conducted using the various combinations of oil, surfactant and cosurfactant, as given in Table 1. No nanoemulsion formation was observed in the 1st (cinnamon oil, Labrasol^®^ and Transcutol HP) and 4th set (Labrafac PG^®^, Labrasol^®^ and Carbitol^®^) with varying ratios of oil:S_mix_ and S_mix_ alone. The 2nd (cinnamon oil, tween 80 and Carbitol^®^) and 3rd set (cinnamon oil, tween 80 and ethylene glycol) yielded nanoemulsion; however, the nanoemulsion region occupied by the 2nd combination was greater than the 3rd set. The ternary phase diagrams depict the ability of surfactant and cosurfactant to solubilize the oil phase to obtain a clear nanoemulsion. Larger nanoemulsion regions indicate maximum capability to form a nanoemulsion with the drug. Figure 1 and Figure 2 show the ternary plots for nanoemulsions obtained using cinnamon oil, tween 80 and ethylene glycol; and cinnamon oil, tween 80 and Carbitol^®^, respectively. In cinnamon oil, tween 80 and ethylene glycol combination, as the amount of tween 80 was increased, a larger nanoemulsion region was observed, as seen in Figure 1 with S_mix_ 4:1. This may be attributed to the better surface coverage of tween 80 over the nanoemulsion droplets leading to a reduction in interfacial tension and leading to spontaneous dispersion [30]. A similar result was observed with cinnamon oil, tween 80 and Carbitol^®^ combination. A large nanoemulsion region was observed with S_mix_ 5:1. Based on the ternary plots, the 2nd set composition of cinnamon oil, tween 80 and Carbitol^®^ containing the S_mix_ ratio of 5:1 and an oil:S_mix_ ratio 1:9 were chosen for optimization. Representative formulation titration trials conducted for the S_mix_ ratio 1:9 and the oil:S_mix_ ratio 5:1 are shown in Table 1.

### 2.3. Optimization of Quercetin Nanoemulsion

Quercetin was incorporated in the nanoemulsion composition optimized based on ternary plots. The amount to be incorporated was based on Minimum Inhibitory Concentration (MIC) study. The MIC study was performed to assess the effectiveness of Quercetin against two prominent periodontal pathogens, namely *Tannerella forsythia* and *Porphyromonas gingivalis*. Based on the MIC study against the periodontal pathogens as given in Table 2, the dose of Quercetin was selected in the range of 75–125 µg/mL. The optimized composition of nanoemulsion was 4.68 mg Quercetin, 125 mg cinnamon oil, 1125 mg tween 80, 225 mg Carbitol^®^, and 6000 mg water. The nanoemulsion was transparent and easily flowable.

### 2.4. Thermodynamic Stability Testing of the Nanoemulsion

A thermodynamic stability study was carried out to assess the kinetic stability of the nanoemulsion. Stress studies such as centrifugation, heating-cooling cycle, and freeze-thaw cycle were performed. The nanoemulsion was found to be stable, and no phase separation or Ostwald ripening were observed during these studies.

### 2.5. Characterization Results for Nanoemulsion

Globule size, Polydispersity Index (PDI) and zeta potential of the nanoemulsion was determined at 10 times and 100 times dilution. Consistent results were observed after both the dilutions. The results of a few selected batches are shown in Table 3. As the amount of oil increased, there was an increase in the globule size [31], whereas zeta potential was found to increase with an increase in the quantity of S_mix_, as the charge on the surface of globules is governed by the presence of surfactants which results in better stabilization of the nanoemulsion. The negative sign of zeta potential may be due to the presence of anionic groups of fatty acids in the cinnamon oil, tween 80 and –OH group of Carbitol^®^. The higher the zeta potential, the higher the stability of the nanoemulsion in general. However, as the nanoemulsion was incorporated in to the gel, the mobility of globules is restricted, and therefore no significant influence of zeta potential can be observed. A decrease in the amount of S_mix_ was found to increase the globule size. This may be attributed to the fact that a higher amount of surfactant is required to stabilize and produce fine droplets of nanoemulsion [30,32]. There was no significant difference in the PDI of nanoemulsions between the batches (*p* > 0.1).

### 2.6. Surface Morphology of Nanoemulsion Using TEM

**Figure 3:**
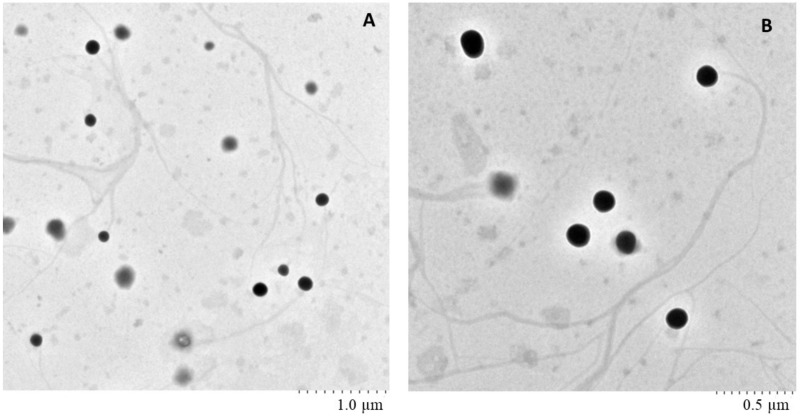
TEM images of nanoemulsion at different magnification (**A**) scale 1.0 µm, (**B**) scale 0.5 µm.

To study the morphology and distribution of nanoemulsion, transmission electron microscopy photomicrographs were taken, as shown in Figure 3. It was observed that the globule size was less than 200 nm, and the globules were discrete and spherical without any aggregation.

### 2.7. Preparation of Nanoemulgel

Various FDA Inactive Ingredient Guide (IIG)-approved polymers were investigated for the in situ gelation process. Poloxamer 188 and poloxamer 407 solutions were prepared separately in concentrations ranging from 15% *w*/*v* to 25% *w*/*v* and checked for in situ gelation at 37 °C. Xanthan gum was tested for in situ gel formation at a concentration ranging from 0.05–0.1% *w*/*v* to 1–3% *w*/*v* utilizing calcium chloride dihydrate and trisodium citrate as crosslinking agents. Gellan gum solutions were prepared at concentration ranges of 0.3–0.7% *w*/*v*. Carbopol 934P was tested for gelation at a concentration range of 0.5% *w*/*v* in combination with 25% *w*/*v* of poloxamer 407 solution. However, no gel formation was observed in the case of xanthan gum, gellan gum and carbopol 934P at 37 °C at any of the concentrations. Hence the polymer poloxamer 407 at a concentration of 23% *w*/*v* was chosen as a suitable gelling agent for in situ gel formation.

Based on the ternary plots, S_mix_ ratio 5:1 and oil:S_mix_ ratio 1:9 was selected as an optimized formula. The aqueous media, water was replaced with the incorporation of an aqueous solution of gel base 23% *w*/*v* poloxamer 407 while preparing nanoemulgel. The optimized composition of nanoemulgel was 4.68 mg Quercetin, 125 mg cinnamon oil, 1125 mg tween 80, 225 mg Carbitol^®^, 1380 mg poloxamer 407, 6000 mg water. 200 µL is the estimated volume that can be instilled inside the periodontal pocket. Accordingly, every 200 µL contained a specified dose, 125 µg of Quercetin.

### 2.8. Characterization Results of Nanoemulgel

In the sol-gel transition study, it was observed that in situ gel formation occurred in 53 ± 2 s at 37 °C. The nanoemulsion passed the syringeability test. The pH of Quercetin nanoemulgel was found to be 6.34 ± 0.5 measured using a pH paper which was in accordance with the pH of the gingival crevicular fluid indicating suitability for drug delivery into the periodontal pocket. The viscosity of the nanoemulgel was found to be low at room temperature, and a drastic increase was observed at body temperature with respect to the gelation capacity of the polymer. Viscosity was 408.3 ± 0.5 cPs at 26 ± 0.5 °C and 30,647.5 ± 0.3 cPs at 37 ± 0.5 °C, demonstrating the sol-gel nature of the formulation. The drug content of the prepared nanoemulgel was found to be 99.5 ± 0.02%. There was no loss of drug during the preparation of nanoemulgel [33,34].

### 2.9. In Vitro Diffusion of Quercetin from Nanoemulgel

**Figure 4:**
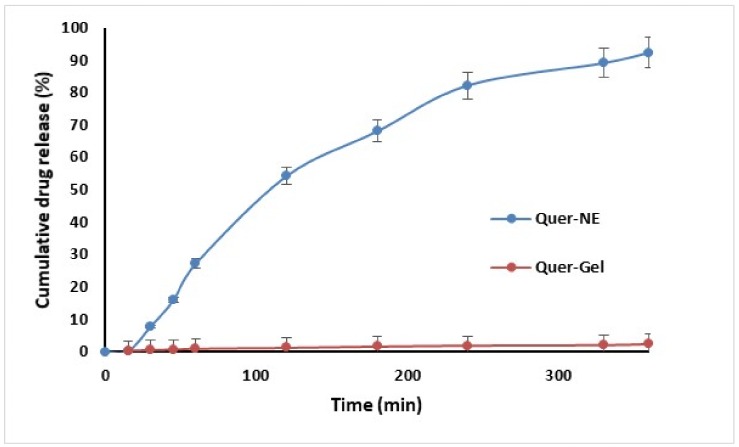
In vitro drug diffusion profile.

**Figure 5:**
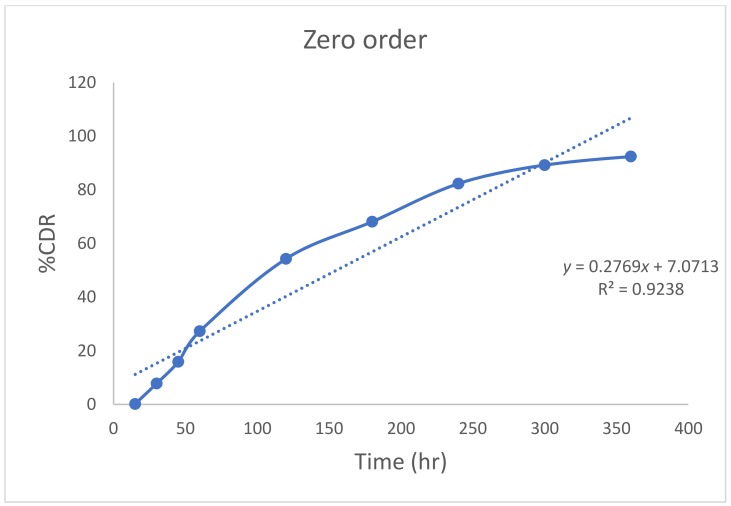
Zero-order release kinetics for nanoemulgel.

The diffusion study of optimized nanoemulgel was carried out in comparison to a gel incorporated with pure Quercetin. The % drug diffused from nanoemulgel was sustained and found to be 92.4 ± 4.6% at the end of 6 h. However, the drug release from the Quercetin-loaded gel (comprising of 23% poloxamer 407 gel loaded with Quercetin) was much less (<3% drug at the end of 6 h), which may be due to the poor solubility of the drug in an aqueous gel base. The higher surface area due to smaller globule size in nanoemulsion permitted higher drug release from nanoemulgel. The sustained drug release from the in situ gel may be due to the matrix structure of the gel, which may be the result of increased micelles within the gel structure. The greater number of cross-links between neighboring micelles results in higher viscosity and a lower rate of drug release [35] (Figure 4). A zero-order release pattern was observed with the formulation (R^2^ = 0.9238) (Figure 5).

### 2.10. Stability Study Results

The formulated nanoemulgel exhibited good physical stability over a storage period of 3 months (90 days). The samples had a pale yellow-colored appearance with a pH ranging from 6 to 6.5. The drug content was more than 97 ± 1.5% for all the time points. The stability data is given in Table 4.

### 2.11. Results of Molecular Dynamic Simulations

**Figure 6:**
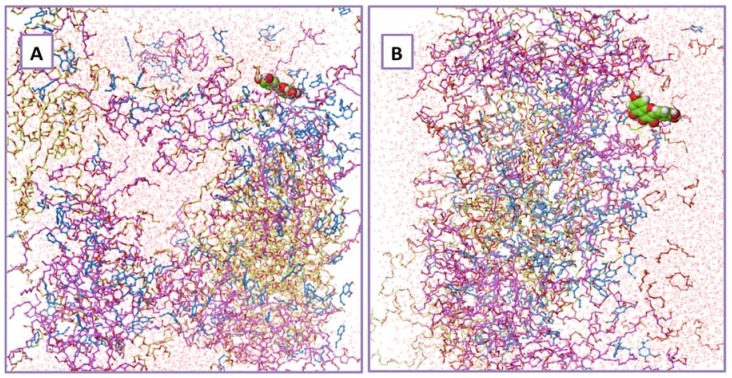
Formation of gel inside the water for the formulation used in this study. (**A**) The formulation components at 275 K; and (**B**) the formulation components at 313 K. Quercetin is represented in the form of a CPK model with green color, whereas the cinnamon oil is represented in cyan color, Carbitol^®^ is represented in pink color, tween 80 is represented in brown color and poloxamer 407 is represented with yellow color ball and tube.

**Figure 7:**
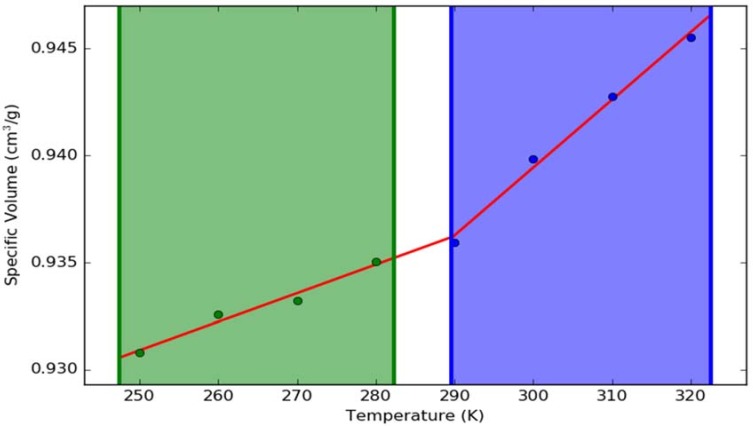
Effect of temperature on the specific volume of the simulation box containing formulation components and water.

The physical properties of the systems at 275 K (1.85 °C) and 313 K (39.85 °C) are presented in Table 5. These property differences at the molecular level provide an insight into the gel phase at 313 K. It has already been reported in the literature that the self-diffusion coefficient of water at 275 K is ~1.2765 × 10^−9^ m^2^/s and the same at 313 K is 3.222 × 10^−9^ m^2^/s [36]. It is evident from the table that the water is more or less in its bulk phase at 275 K, as the formulation components are solvated inside the water phase. On the other hand, the negative difference between the bulk water diffusion coefficient and the same of water in the formulation at 313 K proves slow dynamics of the water (Figure 6). The increase in the number of hydrogen bonds between the formulation components and the water molecules also indicates the increased interaction between the components. Figure 7 exhibits the specific volume change as a function of temperature for the entire formulation mixture. It is evident from the plot that there is a sol to gel phase transformation at ~290 K. This is in very good agreement with the experimental gelation temperature.

## 3. Materials and Methods

### 3.1. Materials

Quercetin was purchased from Himedia, Mumbai. Poloxamer 407, Poloxamer 188 were purchased from Sigma Aldrich, Bangalore, India. Labrafac PG^®^, Labrasol^®^, Carbitol^®^, Transcutol HP^®^ and Capryol 90^®^ were obtained as gift samples from Gatte Fosse, Mumbai, India. Cremophor EL^®^, Lutrol E400, Carbopol 934P, and Lutrol E600 were obtained as gift samples from BASF Corporation, Mumbai, India. Xanthan gum and Gellan gum were purchased from Triveni Chemicals, Vapi, Gujarat, India. Cinnamon oil and Palm oil were purchased from Genuine Chemical Co., Mumbai. Triacetin, Isopropyl myristate and Castor oil was from Himedia Laboratories Pvt. Ltd., Mumbai, India. Sesame oil was from National Chemicals, Gujarat, India. Migliyol was from Peter Cremer, Hamburg, Germany. Tween 80 and Tween 60 were purchased from Nice chemicals Pvt. Ltd., Kochi and Sisco Research Laboratories Pvt. Ltd., Mumbai, India. Tween 20 and Glycerol were from Merck Specialities Pvt. Ltd., Mumbai, India respectively. Span 80 was from Suvidhinath Laboratories, Baroda. Ethylene glycol was procured from Loba Chemie Pvt. Ltd., Mumbai, India. All other chemicals and reagents were of laboratory grade.

### 3.2. Methods

#### 3.2.1. Screening of Nanoemulsion Components

**Figure 8:**
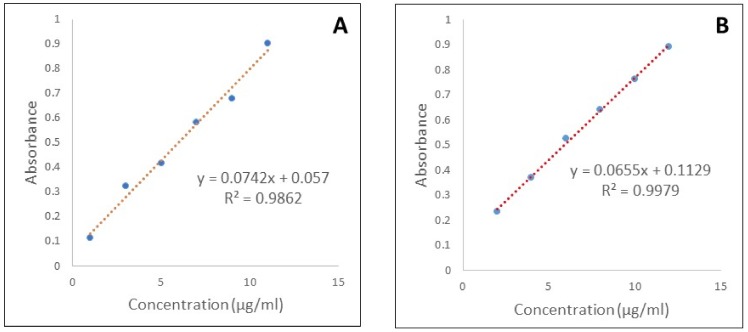
Calibration curve of Quercetin in (**A**) methanol, and (**B**) phosphate buffer pH 7.4.

Screening of the components was carried out based on the saturation solubility of Quercetin in various oils, surfactants, and cosurfactants. The study was carried out by dissolving the excess amount of the drug in 1 mL of oil, surfactant, and cosurfactant respectively. The samples were placed in a water bath shaker (Remi Sales and Engineering Ltd., Mumbai, India) at 37 °C for 72 h. After 72 h, the samples were centrifuged at 10,000 rpm for 5 min (Tarsons, West Bengal, India) and the supernatants were filtered through membrane filters (0.45 µm, Merck Millipore, Burlington, MA, USA), diluted with methanol and analyzed by UV spectrophotometry at 269.2 nm [35,37]. The standard plot is shown in Figure 8.

#### 3.2.2. Construction of Pseudo-Ternary Phase Diagrams

On the basis of the saturation solubility results, cinnamon oil and Labrafac PG^®^ as the oil phase, tween 80 and Labrasol^®^ as a surfactant, and Transcutol HP, ethylene glycol and Carbitol^®^ as cosurfactant were selected. The nanoemulsion was prepared using a spontaneous emulsification method. The formulation was categorized based on the appearance as given in Table 6. Different combinations of oil, surfactant, and cosurfactant were attempted (see Supplementary Materials Table S1). Varying ratios of surfactant-cosurfactant (S_mix_) and oil to S_mix_ were taken, and a specified quantity of water was added by aqueous titration method. The percentage of each component was calculated in the ratios that yielded nanoemulsion, and ternary plots were constructed accordingly using CHEMIX School 3.6 software (Arne Standnes, Norway). The ratios which generated plots that occupied the maximum nanoemulsion region were chosen for further optimization.

#### 3.2.3. Preparation of Quercetin Nanoemulsion 

Based on the region occupied in the pseudo-ternary phase diagrams, the optimum ratios of oil, surfactant, and cosurfactant were chosen. Quercetin was incorporated into the mix to obtain nanoemulsion. The dose of the formulation was selected according to the Minimum Inhibitory Concentration (MIC). MIC of the Quercetin was determined against two prominent pathogens, namely *Porphyromonas gingivalis* and *Tannerella forsythia*, by disc diffusion method. The study was carried out in Maratha Mandal Dental College, Belgaum, Karnataka, India as per the procedure mentioned by Patil et al. [38].

#### 3.2.4. Thermodynamic Stability Testing of the Nanoemulsion

The optimized nanoemulsion was subjected to centrifugation at 3500 rpm for 30 min and observed for cracking, phase separation or any other physical changes. The effect of temperature on the stability of nanoemulsion was observed at different storage temperatures by subjecting to the heating-cooling cycle. The nanoemulsion was stored at 4 °C and 45 °C for not less than 48 h for six cycles and observed for physical stability. The nanoemulsion was also subjected to freeze-thaw cycle at −21 °C and +25 °C with storage of not less than 48 h and observed for physical changes [31].

#### 3.2.5. Globule Size, Polydispersity Index (PDI) and Zeta Potential

The mean globule size and size distribution of nanoemulsion, polydispersity index (PDI) and zeta potential were determined using Malvern Zeta Sizer (Nano ZS, Malvern, UK). The measurements were carried out at 25 °C by dynamic light scattering technique. The zeta potential was measured by laser Doppler velocimetry and phase analysis light scattering technique. 100 µL of nanoemulsion was taken and diluted with Milli-Q water to yield 10 times and 100 times dilution. The diluted samples were subjected to particle size and zeta potential analysis.

#### 3.2.6. Transmission Electron Microscopy (TEM)

Transmission Electron Microscopy (TEM) of nanoemulsion was studied using Hitachi H-7650, Tokyo, Japan. The nanoemulsion was stained with uranyl acetate and dried over copper grids. The grid was examined under TEM.

#### 3.2.7. Preparation of Nanoemulgel

Screening of various polymers such as xanthan gum, gellan gum, carbopol 934P, poloxamer 188 and poloxamer 407 was attempted for the in situ gelation property and polymer showing the optimum gelation time within 1 min was chosen as the appropriate gelling agent. Initially, polymeric solutions were prepared in water at different concentrations. The solutions were stored at 2–8 °C to ensure complete miscibility. The drug Quercetin was dissolved in oil:S_mix_ to obtain a mixture. This mixture was added dropwise to the polymeric solution or the sol system (instead of the aqueous phase in nanoemulsion) with continuous stirring to ensure the homogenous mixing of the oil phase in polymeric solution to form a nanoemulgel and stored at 2–8 °C.

#### 3.2.8. Characterization of Nanoemulgel

The prepared nanoemulgel was evaluated for gelation/gelling time, syringeability, pH, viscosity and drug content.

- Sol-gel transition and syringeability
  The nanoemulgel was observed for in situ gelation or sol-gel transition by the test-tube inverting method as per Srivastava et al. with slight modification [33]. 5 mL of sol stored at 2–8 °C was taken in a test tube, immersed in a water bath (Remi Equipment Ltd., Bangalore, India) maintained at a temperature of 37 °C. The sample was observed for gelation by tilting the test tubes at 90°. Gelation was said to have occurred when the meniscus would no longer move upon tilting and the time taken to gel was recorded. For testing syringeability, nanoemulsion stored at 2–8 °C was filled into a 1 mL syringe with a 22 gauge needle, and gentle force was applied by pressing the piston. The ease of syringeability was observed visually.
- Drug content
  For determining the drug content, 10 mg of the Quercetin nanoemulgel formulation was weighed and dissolved in phosphate buffer pH 7.4. The solution was filtered using Whatman filter paper (No. 41) and suitably diluted. The absorbance of the resulting solution was measured by UV spectrophotometry (UV-160 Shimadzu Corporation, Kyoto, Japan) at 269.2 nm and the drug content was determined.
- pH measurement
  The pH of the nanoemulgel was read using a pH paper owing to the consistency of the formulation.
- Viscosity determination
  The viscosity was determined using Brookfield DV III ultra-programmable rheometer (Brookfield Engineering Laboratories, Middleboro, MA, USA). The viscosity of Quercetin nanoemulgel was measured at 26 °C and 37 °C, respectively. The instrument was calibrated using spindle 40 with viscosity standard fluid prior to the sample measurements.

#### 3.2.9. In Vitro Diffusion Study and Release Kinetics

The in vitro drug diffusion study was performed using Franz vertical diffusion cells (Orchid Scientific & Innovative India Pvt. Ltd., Maharashtra, India). The receptor compartment was filled with phosphate buffer pH 7.4 solution and was kept in contact with the dialysis sac (MW 6000–8000 cut-offs, Sigma Aldrich, Saint Louis, MO, USA). The semipermeable membrane was firmly sandwiched between the donor and the receptor compartments. 200 µL of Quercetin nanoemulgel was placed on the semipermeable membrane. 20 mL phosphate buffer pH 7.4 was placed in receptor compartment. The Franz diffusion cell apparatus was integrated with a temperature regulating water bath to maintain a temperature of 37 °C during the diffusion study. The rpm was set at 500 during the process, and 500 µL sample aliquots were withdrawn at regular time intervals. The samples were analyzed using UV spectrophotometry at 269.2 nm. Various mathematical models were used to describe the release kinetics such as zero-order, first-order, the Higuchi and the Korse-Meyer Peppas models [39].

#### 3.2.10. Stability Study

Quercetin nanoemulgels were subjected to stability study as per ICH guidelines. The samples were stored in amber-colored crimped glass vials at 25 °C ± 2 °C/60% RH ± 5% RH in stability chambers (Thermolab, Mumbai, India). The control samples were stored at refrigeration temperature (2–8 °C). The samples were evaluated at specified time intervals for their appearance, pH and drug content.

#### 3.2.11. Molecular Dynamics (MD) Simulations

Molecular dynamics simulations mimicking both the solution and gel phase was carried out using Schrödinger—Materials Science Suite (MS-Suite) version 2018-1. The representative stoichiometric ratio of all the components in the formulation was packed as an amorphous system inside a simulation box using disordered system builder within the framework of MS-Suite (See Table 7 for the component details). The following material relaxation protocol was used to build an equilibrated sample at necessary temperatures. Brownian dynamics (BD) for 10 ps at 10 K under NVT conditions followed by 100 ps BD at 100 K under NPT ensemble. These BD simulations allowed the systems to naturally evolve output of packing effects and achieve the force field defined Van der Waals volume. Subsequently, a 100 ps MD simulations were carried out under NPT conditions using an anisotropic pressure coupling at 300 K. Finally, a 1200 ps of MD simulation was carried out either at 275 K or 313 K under NPT conditions.

The well-equilibrated samples at 275 K and 313 K were chosen for the production run of 100 ns, and the trajectory was recorded at every 100 ps time interval. Unless otherwise mentioned, the rest of the parameters were set to default values in the multistage workflow of MS-Suite. The following analysis was carried out in the final 800 frames of the production run. To calculate the effect of temperature on the gelation process, a separate MD simulation protocol was used under the thermophysical properties workflow of MS-Suite. The initial and final temperature range was set to 250, and 320 K with the step values of 10 K. 10 ns simulation with 3 individual systems were carried out to get the specific volume of the system.

## 4. Conclusions

The developed nanoemulgel of Quercetin using cinnamon oil, tween 80, Carbitol^®^ and poloxamer 407 exhibited good physical characteristics, stability, sol-gel transition, and syringeability. MD simulation showed specific volume change in the formulation mixture as a function of temperature. It was evident from the computational study that there is a sol to gel phase transformation. Thus, in vitro studies proved that the nanoemulgel of Quercetin formulation could be successfully used in periodontitis. However, the preclinical and clinical efficacy of the formulation needs to be established in vivo.

## Figures and Tables

**Table 1:** Formulation titration trials conducted for S_mix_ ratio 1:9 (Tween 80:Carbitol^®^) and 5:1 (Cinnamon oil:S_mix_ ratio).

Oil (mg)	S_mix_ (mg)	Water (mg)	Water Added	Total (mg)	Appearance	Formulation	Oil (%)	S_mix_ (%)	Water (%)
20	180	30	30	230	Transparent, easily flowable	Nanoemulsion	8.69	78.2	13.04
20	180	90	60	290	Transparent, easily flowable	Nanoemulsion	6.89	62.0	31.03
20	180	190	100	390	Transparent, easily flowable	Nanoemulsion	5.12	46.15	48.71
20	180	390	200	590	Transparent, easily flowable	Nanoemulsion	3.38	30.50	66.10
20	180	590	200	790	Transparent, easily flowable	Nanoemulsion	2.53	22.78	74.68
20	180	1090	500	1290	Transparent, easily flowable	Nanoemulsion	1.55	13.95	84.49
20	180	1590	500	1790	Transparent, easily flowable	Nanoemulsion	1.11	10.05	88.82

**Table 2:** MIC study of Quercetin against periodontal pathogens.

Q (µg/mL)	100	50	25	12.6	6.25	3.12	1.6	0.8	0.4	0.2
Tf	S	S	R	R	R	R	R	R	R	R
Pg	S	R	R	R	R	R	R	R	R	R

Note: Q—Quercetin; S—Sensitive; R—Resistant; Tf—*Tannerella forsythia*; Pg—*Porphyromonas gingivalis*.

**Table 3:** Globule size, Polydispersity Index (PDI) and Zeta potential of nanoemulsion comprising of tween 80: Carbitol^®^ as S_mix_ and cinnamon oil.

Sr. No.	Sample Name	Z Average Size (nm)	PDI	Zeta Potential (mV)
1	Smix ratio 5:1 Oil: Smix 1:7	153.51 ± 8.23	0.60 ± 0.03	−3.03 ± 0.02
2	Smix ratio 5:1 Oil: Smix 1:9	138.90 ± 10.14	0.56 ± 0.04	−3.58 ± 0.01
3	Smix ratio 4:1 Oil: Smix 1:7	136.05 ± 10.17	0.48 ± 0.07	−9.20 ± 0.02
4	Smix ratio 4:1 Oil: Smix 1:9	125.60 ± 9.42	0.53 ± 0.10	−12.10 ± 0.07
5	Smix ratio 3:1 Oil: Smix 1:7	115.20 ± 8.31	0.47 ± 0.05	−3.16 ± 0.02
6	Smix ratio 3:1 Oil: Smix 1:9	104.33 ± 6.28	0.39 ± 0.12	−4.03 ± 0.04

**Table 4:** Stability assessment data of Quercetin nanoemulgel.

Days	Drug Content	pH	Colour
0	99.98 ± 0.24	6.5	Pale yellow
5	99.72 ± 0.40	6.5	Pale yellow
30	98.98 ± 0.78	6.5	Pale yellow
60	98.12 ± 1.1	6.5	Pale yellow
90	97.0 ± 1.5	6.5	Pale yellow

**Table 5:** Physical properties of the formulation in both sol at 275 K (1.85 °C) and gel phase at 313 K (39.85 °C).

Property	At 275 K	At 313 K
Density	1065 ± 4 kg/m^3^	1060 ± 6 kg/m^3^
No. of Hydrogen bonds between formulation components and solvent	991 ± 20	1200 ± 21
Diffusion coefficient of solvent	1.2504 × 10^−9^ m^2^/s	2.8332 × 10^−9^ m^2^/s
Diffusion coefficient of formulation components	1.0746 × 10^−10^ m^2^/s	2.4066 × 10^−10^ m^2^/s
Radii of gyration of Poloxamer 407 molecules	29.45 ± 2.2	31.72 ± 4.3

**Table 6:** Categorization criteria.

Appearance	Attributed Formulation
Transparent and easily flowable	Nanoemulsion
Transparent and difficult to flow	Nanoemulgel
Milky/Cloudy and easily flowable	Emulsion
Milky/Cloudy and difficult to flow	Emulgel

**Table 7:** Chemical composition of the formulation in MD simulations.

Component	No. of Molecules	Molecular Mass	Composition in mg
Cinnamon Oil	34	282.38	125.00
Quercetin	1	302.24	4.68
Tween 80	55	1310.00	1125.00
Carbitol^®^	108	134.17	225.00
Poloxamer 407	3	31,057.17	1380.00
Water	10,000	18.01	6000

## Supplementary Materials

The following are available online. Figure S1: Saturation solubility profile of Qquercetin in various oils, Figure S2: Saturation solubility profile of Qquercetin in various surfactants and cosurfactants, Table S1: Composition of various formulation trials.
