# Supplementary material for: Localized In Situ Nanoemulgel Drug Delivery System of Quercetin for Periodontitis: Development and Computational Simulations
Source: Molecules. 2018 Jun 4;23(6):1363. doi: 10.3390/molecules23061363 (PMC6099597; doi:10.3390/molecules23061363)
Supplement: Supplementary file 1 [file molecules-23-01363-s001.pdf]

Supplimentory figures and table

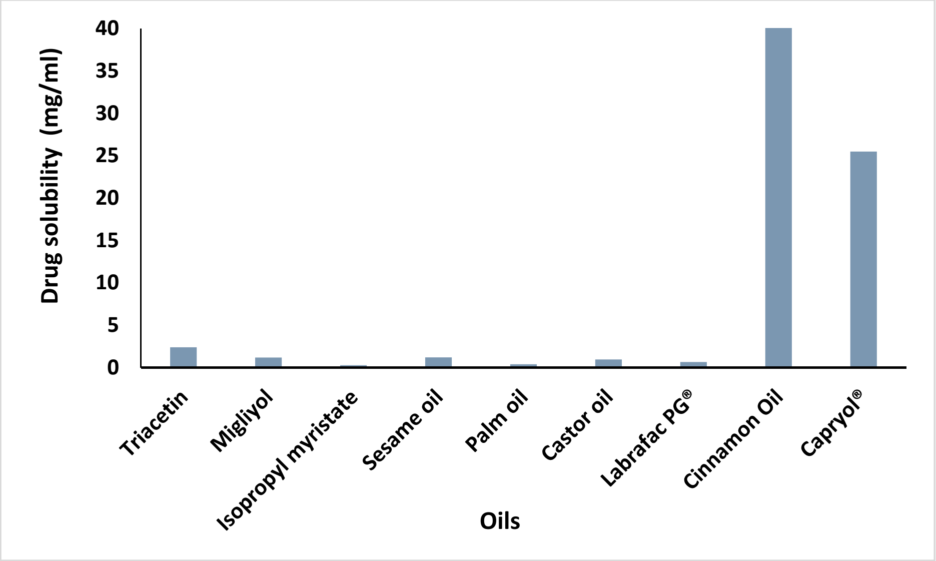

Figure S1. Saturation solubility profile of Quercetin in various oils

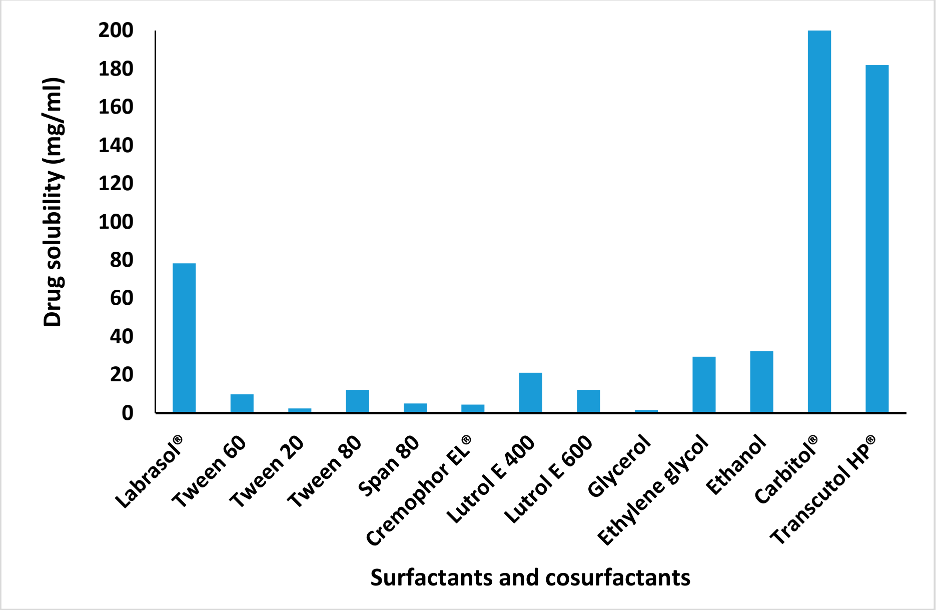

Figure S2. Saturation solubility profile of Quercetin in various surfactants and cosurfactants

Table S1. Composition of various formulation trials

Set No.	Composition	Oil: S_{mix} ratios	Surfactant: Cosurfactant (S_{mix}) ratios
**1**	Cinnamon oil, Labrasol-surfactant and Transcutol HP-cosurfactant	1:1, 1:2, 1:3, 1:4, 1:5, 1:6, 1:7, 1:8, 1:9	1:0, 1:2 and 3:1
**2**	Cinnamon oil, Tween 80-surfactant and Carbitol®-cosurfactant	1:1, 1:2, 1:3, 1:4, 1:5, 1:6, 1:7, 1:8, 1:9	1:3, 3:1, 1:0, 4:1, 5:1
**3**	Cinnamon oil, Tween 80-surfactant and ethylene glycol-cosurfactant	1:1, 1:2, 1:3, 1:4, 1:5, 1:6, 1:7, 1:8, 1:9	1:0, 1:1, 2:1, 1:4, 4:1
**4**	Labrafac PG®-oil phase, surfactant-Labrasol® and cosurfactant-Carbitol®	1:1, 1:2, 1:3, 1:4, 1:5, 1:6, 1:7, 1:8, 1:9	1:0, 1:2, 3:1 and 7:1
